# Targeting Angiogenesis and Visual Cycle in Age-Related Macular Degeneration: The Role of Stem Cells and Vinpocetine

**DOI:** 10.7759/cureus.93878

**Published:** 2025-10-05

**Authors:** Stavroula Almpanidou, Eleni Gounari, Antonios Goulas, Fotios Topouzis, Persefoni Talimtzi, Kokkona Kouzi-Koliakou, Vasileios Karampatakis, George Koliakos

**Affiliations:** 1 Experimental Ophthalmology, Aristotle University of Thessaloniki, Thessaloniki, GRC; 2 Biochemistry, Aristotle University of Thessaloniki, Thessaloniki, GRC; 3 Pharmacology, Aristotle University of Thessaloniki, Thessaloniki, GRC; 4 Ophthalmology, Aristotle University of Thessaloniki, AHEPA Hospital, Thessaloniki, GRC; 5 Experimental Ophthlalmology, Aristotle University of Thessaloniki, Thessaloniki, GRC; 6 Histology and Embryology, Aristotle University of Thessaloniki, Thessaloniki, GRC; 7 Biological Chemistry, Aristotle University of Thessaloniki, Thessaloniki, GRC

**Keywords:** age-related macular degeneration, angiogenesis, arpe-19, bone marrow stem cells, vinpocetine, visual cycle

## Abstract

Purpose

The purpose of this study was to evaluate whether bone marrow stem cells (BMSCs), vinpocetine, or their combination can attenuate amyloid-β (Aβ)-induced alterations in angiogenesis and visual cycle gene expression in a cellular model of age-related macular degeneration (AMD).

Methods

Human retinal pigment epithelium (RPE) cells (ARPE-19) were exposed to Aβ 1-42 for 24h and divided into four groups: (i) co-culture with BMSCs, (ii) treated with vinpocetine, (iii) treated with BMSCs and vinpocetine, and (iv) untreated control. Cell viability was assessed using the Cell Counting Kit-8 (CCK-8) assay. Quantitative real-time polymerase chain reaction (qRT-PCR) was employed to evaluate the mRNA expression levels of angiogenesis-related genes, vascular endothelial growth factor (VEGF-A) and pigment epithelium-derived factor (PEDF), and RPE-associated visual cycle genes: lecithin retinol acyltransferase (LRAT), retinoid isomerohydrolase (RPE65), retinol dehydrogenase 5 (RDH5), retinol dehydrogenase 10 (RDH10), and retinaldehyde-binding protein 1 (RLPB1).

Results

Aβ1-42 significantly reduced ARPE-19 cell viability (p=0.002); all treatments significantly restored viability. Aβ1-42 upregulated VEGF-A expression, which was significantly downregulated by all treatments. Although Aβ1-42 slightly increased PEDF expression, all treatments significantly enhanced its upregulation, with the combination therapy showing the greatest effect (p=0.006, 0.010, and 0.002, respectively). Furthermore, Aβ 1-42 induced upregulation of most visual cycle genes was reversed by all treatments.

Conclusion

Aβ1-42 induces cytotoxicity, angiogenesis, and dysregulation of visual cycle genes in RPE cells in vitro. BMSCs, vinpocetine, and their combination attenuate these effects, supporting a potential role in AMD therapy pending further investigation.

## Introduction

Age-related macular degeneration (AMD) is the leading cause of visual impairment in individuals over 65 in developed countries [1]. AMD is a multifactorial disease characterized by progressive degeneration of retinal pigment epithelium (RPE) and photoreceptors [1]. The RPE, a monolayer of pigmented cells between the neurosensory retina and the choriocapillaris, performs essential functions, including light absorption, phagocytosis, visual cycle maintenance, and angiogenic balance [2]. This delicate epithelium is disrupted by extracellular deposits of matrix proteins and lipids, known as drusen, located between Bruch's membrane and the RPE [3]. Drusen are a hallmark of AMD; their size and number, along with the presence of choroidal neovascularization (CNV), correlate with disease severity [3].

AMD pathophysiology involves genetic and environmental factors along with age-related metabolic processes; yet it is only partially understood. These factors contribute to chronic oxidative stress and inflammation, decline in autophagy [3,4], blood retina barrier (BRB) malfunction, angiogenesis, and dysregulation of the visual cycle [5]. Notably, age-related upregulation of visual cycle genes may cause toxic bisretinoid accumulation, contributing to atrophic AMD [5].

Drusen are also rich in amyloid-β (Aβ) aggregates [6], linking AMD with neurodegenerative processes observed in Alzheimer's disease [7]. Aβ promotes vascular endothelial growth factor-A (VEGF-A) expression and induces RPE apoptosis, facilitating CNV and blood-retinal barrier disruption [6]. Overexpression of VEGF is central to neovascular AMD, while reduced levels of pigment epithelium-derived factor (PEDF), a serine protease inhibitor, exacerbate angiogenesis [8]. The balance between PEDF and VEGF is critical, and its disruption drives CNV development [8].

Current intravitreal anti-VEGF therapies, though effective, have several limitations and adverse effects, highlighting the need for alternative therapeutic strategies [9]. Bone marrow-derived mesenchymal stem cells (BMSCs) have shown potential to modulate the retinal environment and neovascularization [10], with several clinical trials assessing their safety and efficacy (NCT03011541, NCT01736059, NCT03772938, NCT05147701, and NCT02016508) [11]. Interestingly, combining mesenchymal stem cells (MSCs) with anti-inflammatory agents may enhance neural tissue repair [12]. Vinpocetine, a natural NF-κB inhibitor derived from the periwinkle plant, exhibits anti-inflammatory and neuroprotective effects and may complement BMSC therapy by modulating pathogenic pathways in the RPE [13].

In this frame, this study investigates the effect of BMSCs, vinpocetine, and their combination on an Aβ 1-42 induced cellular model of AMD [14,15], focusing on angiogenic factor regulation and visual cycle gene expression. Gene expression analysis is crucial for understanding the molecular mechanisms underlying age-related diseases.

## Materials and methods

Materials and reconstitutions

A human RPE cell line (ARPE-19; ATCC® CRL-2302™) was obtained from the American Type Culture Collection (Manassas, VA, USA). The cells were cultivated in Dulbecco's modified Eagle medium (DMEM; Biowest, Riverside, MO, USA) and F12 medium (Gibco, ANTISEL, Athens, Greece) at a 1:1 ratio. The culture medium was supplemented with 100 units/ml penicillin G (Gibco, ANTISEL, Thessaloniki, Greece), 100 μg/mL streptomycin (Gibco, ANTISEL), and 10% (v/v) fetal bovine serum (FBS; Biowest). 

ARPE-19 is a human retinal pigment epithelial (RPE) cell line that has been widely utilized as an alternative to native RPE due to its maintenance of epithelial cell morphology and its expression of several RPE-specific genes [16]. In our study, all experimental interventions were performed on ARPE19 cells (ATCC® CRL-2302™) at passage four in a 24-well plate since it is known that ARPE-19 cells lose their specialized properties after multiple passages [16,17].

Aβ 1-42 (Anaspec Protein, Fremont, CA) was reconstituted in 2.5% dimethyl sulfoxide (DMSO) in compliance with the manufacturer's guidelines and then diluted to a final concentration of 10 μM in cell culture for 24h incubation, a method previously validated for in vitro modeling of AMD [14,15]. 

Vinpocetine (Y0000589) was purchased from Life Science Chemilab (Athens, Greece). Based on previous data for in vitro tests, vinpocetine was diluted to the required concentration after being dissolved in 2.5% DMSO as a vehicle at a stock concentration of 5 mg/ml [13]. The negative control group was treated with an equivalent amount of 2.5% DMSO (Sigma Aldrich-Merck SA, Athens, Greece), which serves as the solvent for Aβ1-42. 

ARPE-19 culture and measurements of surviving cells by CCK-8 assay

ARPE-19 cells were carefully placed into T-25 flasks for scale-up development. For the experiment, ARPE-19 cells were seeded in 24-well plates at a concentration of 1x105 cells/mL per well and incubated at a temperature of 37°C with 5% CO2. Twenty-four hours after treatment, the Cell Counting kit-8 (CCK-8, Sigma-Aldrich Merck SA) was used to determine the number of viable cells, according to the manufacturer's instructions. Briefly, ARPE-19 cells were incubated with WST-8 (2(2-methoxy-4-nitrophenyl)-3-(4-nitrophenyl)-5-(2,4-disulfophenyl)-2H tetrazolium monosodium salt) solution at 37 °C in the dark for two hours, and the optical density (OD) at 450 nm was measured with PerkinElmer Spectrophotometer Victor 3V (Boston, MA, USA). 

BMSCs isolation, cultivation, and characterization

Bone marrow samples were obtained from donors following their written consent as previously described [18]. Specifically, the study protocol was approved by the Hospital's Review Board and Bioethics Committee of the School of Medicine, Aristotle University of Thessaloniki (3868/24.1.2018). Bone marrow was aspirated under general anaesthesia from both pelvic bones of a 35-year-old male donor. Eligibility assessment followed World Marrow Donor Association criteria. In brief, 5-10 ml of bone marrow was collected in citrate-phosphate-dextrose (CPD) anticoagulant, and after dilution in twice the volume of phosphate-buffered saline (PBS; Biowest), it was reconstituted in half the volume of phicol (Histopaque). The mononuclear cell layer was collected after centrifugation for 20 minutes at 700x G, without brakes. 1.5x106 mononuclear cells (MNCs) were plated in DMEM nutrient solution (DMEM; Biowest) supplemented with 15% fetal bovine serum (FBS; Biowest) and 1% penicillin-streptomycin (penstrep; Sigma Aldrich Merck SA) in a six-well plate, and cells were cultured to 80% confluency in 75cm^2^ tissue culture flasks, in an incubator at 37°C with 5% CO2, with a change of nutrient every two to three days. Between passages two and three, cells were characterized by flow cytometry for the expression of surface markers CD90-CD105-CD29-CD73 and the absence of CD45-CD34. BMSCs were cultivated at a maximum of six passages before any exposure to Aβ 1-42. After induced differentiation for 25-30 days, BMSCs were examined for their ability to differentiate into osteocytes, adipocytes, and chondrocytes using the appropriate media, with media changes occurring every two to three days. Alizarin Red, Oil Red O, and Alcian Blue staining were used, in accordance with the manufacturer's instructions for each differentiation medium, to estimate the success of differentiation.

Treatment with BMSCs 

To investigate the efficacy of BMSCs to attenuate Aβ 1-42-induced changes in vitro, 1x105 BMSCs were placed in the upper chamber of 24-well transwell culture systems (pore size 0.22 μm; Corning Costar, Sigma Aldrich Merck SA). ARPE-19 cells were placed in the lower chambers and reached at least 70-80% confluency. ARPE-19 cells were previously exposed to Aβ1-42 for two hours. After coincubation for 24 hours (37°C, 5% CO2), ARPE-19 cells were gathered and RNA was extracted. Using specially created primers, real-time PCR was used to measure the expression of angiogenesis and visual cycle-related genes.

Treatment with vinpocetine

To assess the effect of vinpocetine on Aβ 1-42-induced changes in vitro, 1μl/ml vinpocetine in 2.5% DMSO was added to ARPE19 cells (ATCC® CRL-2302™) at passage four in a 24-well plate. The cells were previously exposed to Aβ 1-42 for two hours. ARPE-19 cells were incubated at 37 °C, in 5% CO_2_ for 24h, and collected for further analysis. 

Treatment with BMSCs-vinpocetine

To examine the efficacy of the combination of BMSCs with vinpocetine to attenuate the Aβ 1-42-induced changes, ARPE-19 cells were placed in the lower chambers of a transwell culture system, and 1μl/ml vinpocetine in 2.5% DMSO was added. After a coincubation of two hours, 1x105 BMSCs were placed in the upper chambers of the 24-well transwell culture system, and the ARPE-19 cells were incubated at 37 °C, in 5% CO2 for 24h.

VEGF-A and PEDF messenger RNA induction

After 24 hours of incubation with Aβ 1-42 with or without treatment, the High Pure RNA Tissue Kit (Sigma Aldrich-Merck SA) was used to extract total RNA from ARPE-19 cells according to the manufacturer's protocol. The PCR primers corresponding to nucleotides 2465-2536 of the mRNA of human VEGF-A (NM_001025366) and 489-630 for PEDF mRNA (NM_002615) were synthesized as previously described [6]. Real-time reverse transcription polymerase chain reaction (RT-PCR) was performed using KAPA SYBR® Premix Ex Taq™II (Sigma Aldrich-Merck SA) in a PCR cycler Rotor Gene 6000 Real-Time PCR.

Visual cycle genes messenger RNA induction

Following 24 hours of incubation with Aβ 1-42 with or without treatment, total RNA was extracted from ARPE-19 cells using the High Pure RNA Tissue Kit (Sigma Aldrich-Merck SA) in accordance with the manufacturer's protocol. The PCR primers corresponding to nucleotides of the mRNA of human lecithin retinol acyltransferase (LRAT): 5'-TGCGAGCACTTCGTGACCTACT-3', retinoid isomerase (RPE65): 5'-TTTGGCACCTGTGCTTTCCCAG-3', retinol dehydrogenase 5 (RDH5): 5'-CTGTGACCAACCTGGAGAGTCT-3', retinol dehydrogenase 10 (RDH10): 5'-ACGCAGAGCAATGAGGAGAC-3', and retinaldehyde-binding protein 1 (RLPB1): 5'-CCTACAATGTGGTCAAGCCCTTC-3' were synthesized as previously described [17]. Real-time reverse transcription polymerase chain reaction (RT-PCR) was performed using KAPA SYBR® Premix Ex Taq™II (Sigma Aldrich-Merck SA) in a PCR cycler Rotor Gene 6000 Real-Time PCR.

Statistical analysis

Means and standard error of the means (SEM) were used to express the results. The independent samples t-test was used to evaluate the differences in VEGF-Α, PEDF, retinol dehydrogenase 5 (RDH5), retinol dehydrogenase 10 (RDH10), RPE65, lecithin retinol acyltransferase (LRAT), and LRP1b mRNA expression and OD at 450nm between the different groups. Statistical analysis was performed using SPSS version 28 (IBM Inc., Armonk, New York), and a statistical significance level was set at p-value ≤ 0.05.

## Results

Isolation of BMSCs

**Figure 1:**
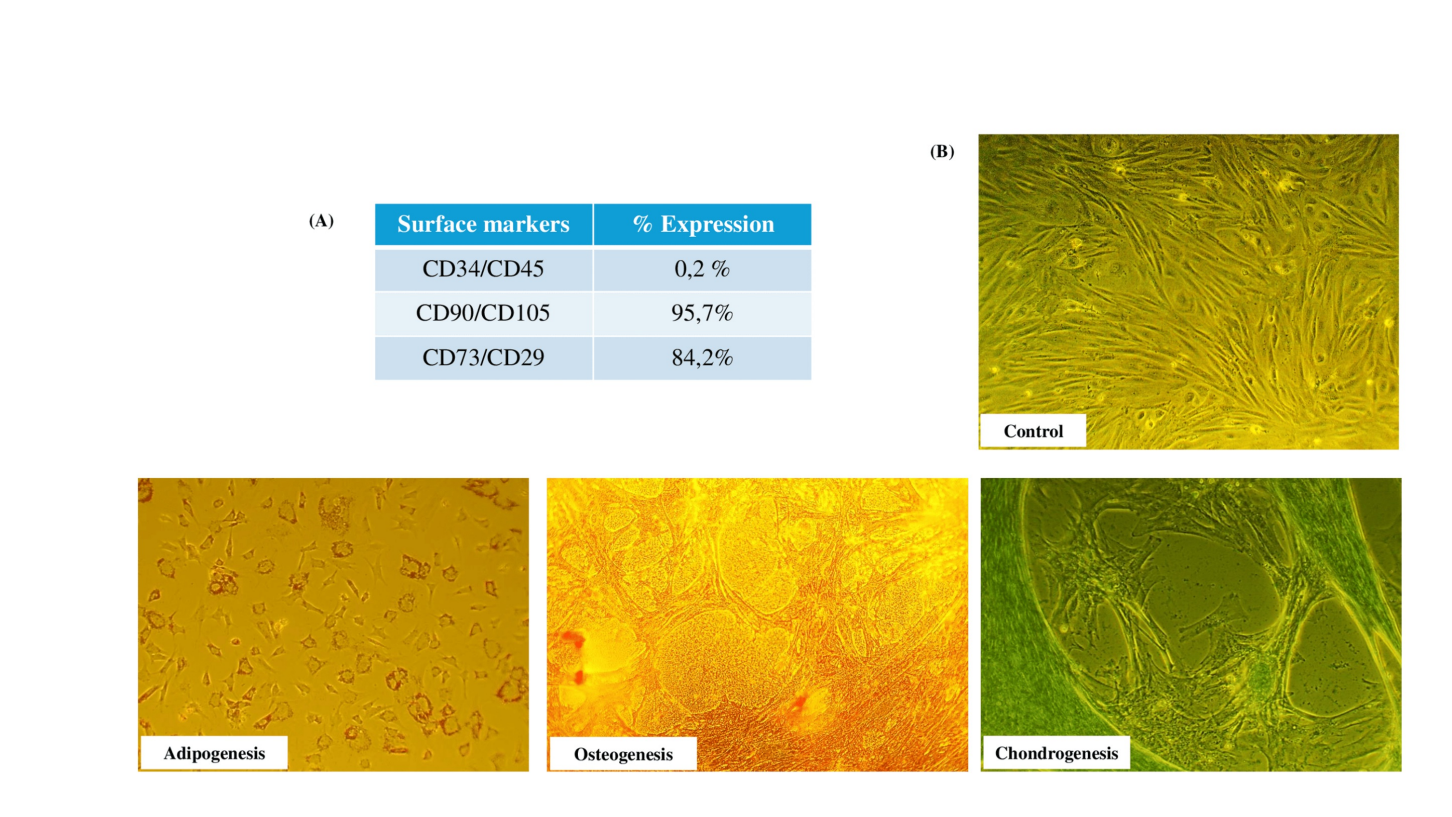
Immunophenotype and differentiation potential of BMSCs A) Immunophenotypic characterization of isolated BMSCs at passage three presented as % expression of the most common mesenchymal stem cells (MSCs) surface markers as defined by flow-cytometry. B) Morphological depiction of bone marrow stem cells (BMSCs) differentiation capacity after specific staining towards adipocytes, osteocytes, and chondrocytes in comparison with undifferentiated control cells. The images were acquired using a 10× objective lens

Flow cytometry analysis was performed to characterize the surface markers of BMSCs, as illustrated in Figure 1A. The absence of hematological marker expression (CD45/CD34) and the high co-expression percentage of CD90/CD105 and CD73/CD29 markers verified the successful isolation of a purified BMSCs population. After labeling with the appropriate dyes, the ability of BMSCs to differentiate into adipocytes, osteocytes, and chondrocytes was verified in comparison to the control-undifferentiated group, as shown in Figure 1B. 

Cell viability 

**Figure 2:**
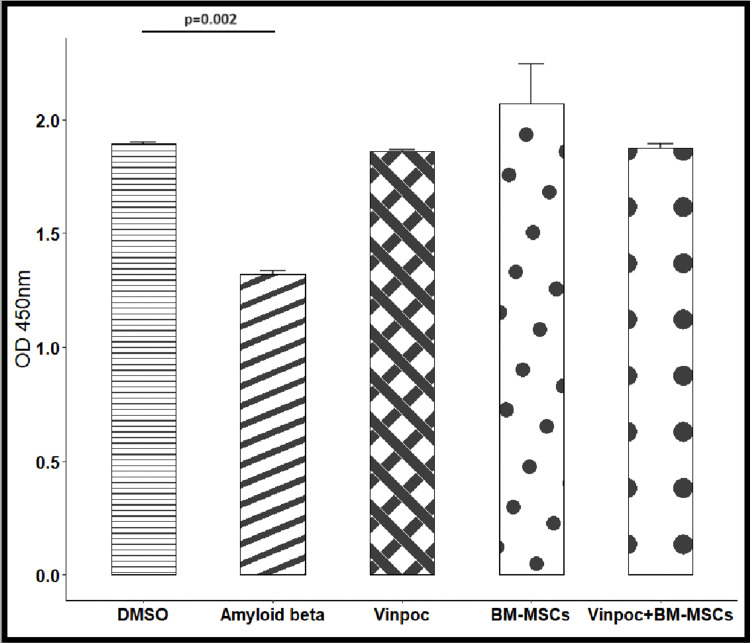
Protective effects of BMSCs and vinpocetine on Aβ-induced cytotoxicity in ARPE-19 cells The ARPE-19 cells were treated with 2.5% DMSO (control group), 10 μM Aβ 1–42, 1x105 BMSCs, 1μl/ml vinpocetine, and their combination, and the number of living cells was determined by the CCK-8 assay. The number of living cells was decreased by exposure to 10 μM of Aβ 1–42 compared to the control group, but was essentially unaffected upon subsequent addition of BMSCs, vinpocetine, and their combination. Bars represent means ± SEM (whiskers); n=5 for each group. BMSCs - bone marrow stem cells; DMSO - dimethyl sulfoxide; CCK-8 - Cell Counting Kit; SEM - standard error

Aβ 1-42 significantly reduced ARPE-19 cell viability compared to the control group (Table 1). However, treatment with BMSCs, vinpocetine, and their combination (vinpocetine+BMSCs) effectively restored cell viability, with the number of viable cells approaching levels observed in the control group (Figure 2).

**Table 1:** ARPE-19 cell viability in the different groups of intervention ^a ^Compared to 2.5% DMSO control; *Statistically significant DMSO - dimethyl sulfoxide; BMSCs - bone marrow stem cells; SD - standard deviation; SEM - standard error

	Mean	SD	SEM	T-test p^a^	Cell Viability(%)
DMSO	1.894	0.011	0.008		100.00
Αβ1-42	1.32	0.021	0.015	0.002*	69.69
Vinpocetine	1.861	0.009	0.0065	0.077	98.23
BMSCs	2.071	0.248	0.1754	0.419	109.32
Vinpocetine+BMSCs	1.875	0.028	0.0195	0.224	98.97

Aβ1-42-induced changes in the level of mRNA of VEGF-A and PEDF in ARPE-19 cells

**Figure 3:**
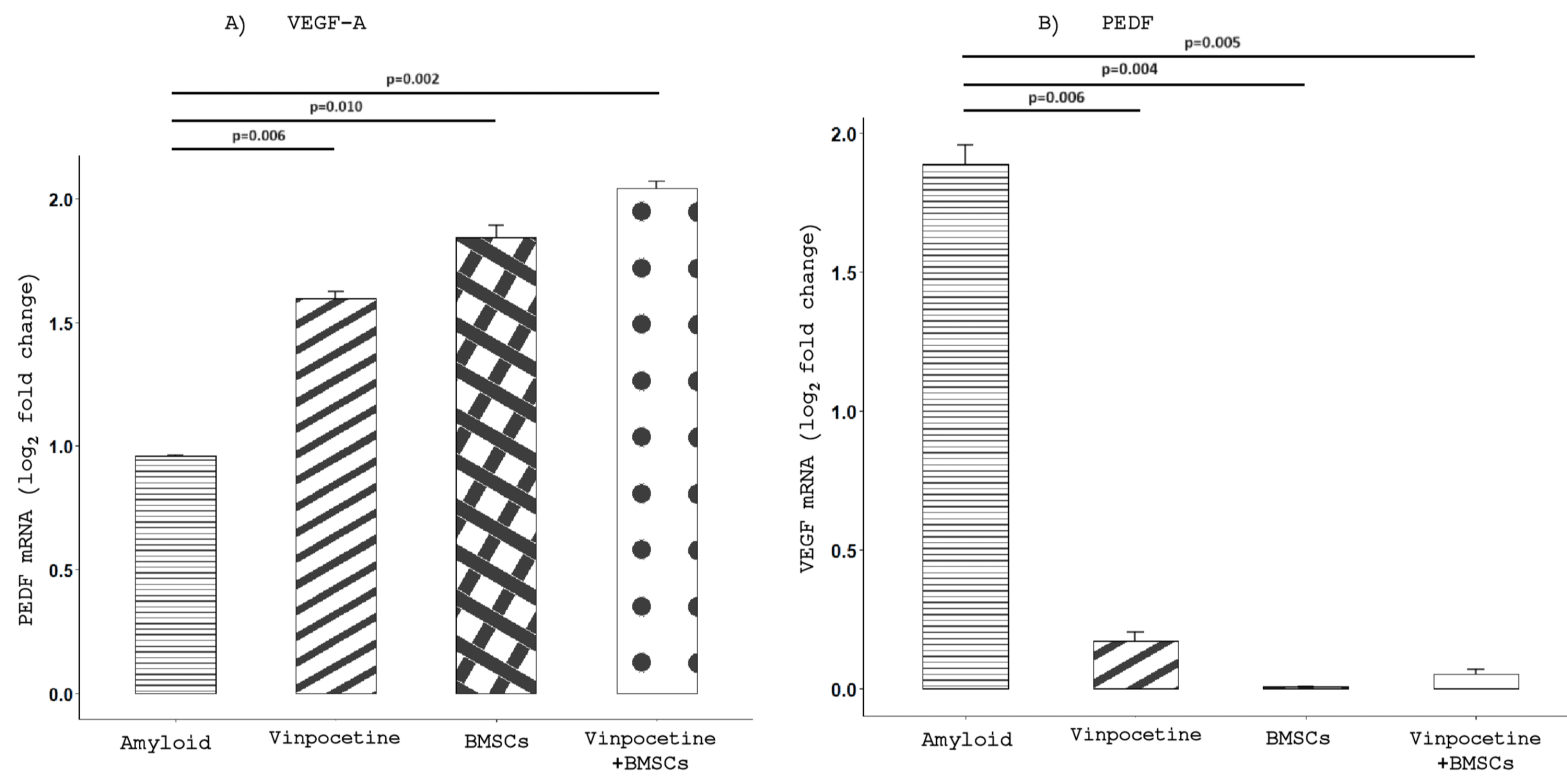
Modulation of VEGF-A and PEDF mRNA expression by BMSCs and vinpocetine in Aβ₁₋₄₂-treated ARPE-19 cells A) VEGF-A mRNA expression in ARPE-19 cells upon exposure to 10 μM Aβ1-42 and subsequent exposure to vinpocetine, to BM-MSCs, and to vinpocetine+BMSCs. VEGF-Α mRNA expression for each group was standardized based on the DMSO control group. Bars represent means ± SEMs (whiskers); n=4 for each group. B) Induction of PEDF mRNA expression by exposure to 10 μM Aβ1-42 and further increase following the exposure to vinpocetine, to BΜSCs, and to their combination (vinpocetine+BMSCs). PEDF mRNA expression for each group was standardized based on the DMSO control group. Bars represent means ± SEMs (whiskers); n=4 for each group. VEGF-A - vascular endothelial growth factor; PEDF - pigment epithelium-derived factor; BMSCs - bone marrow stem cells; SEM - standard error; DMSO - dimethyl sulfoxide

The level of expression of the mRNA of VEGF-A was determined by real-time RT-PCR (Table 2). A significant upregulation of VEGF-A was observed exclusively in the group exposed to 10 μM Aβ 1-42. Subsequent treatment with vinpocetine, BMSCs, and their combination (vinpocetine+BMSCs) markedly reduced VEGF-A mRNA levels, reversing the Aβ 1-42-induced increase (Figure 3A).

**Table 2:** VEGF-A and PEDF mRNA level expression in the ARPE-19 cells in the different groups of intervention VEGF-A and PEDF mRNA expression for each group was standardized based on the 2.5% DMSO control group, a comparison of each group with Amyloid beta; * indicated statistical significance VEGF-A - vascular endothelium growth factor-A; PEDF - pigment epithelium-derived factor

	VEGF-A				PEDF			
	Mean	SD	SEM	T-test p	Mean	SD	SEM	T-test p-value^a^
Αβ1-42	1.887	0.102	0.072		0.959	0.009	0.006	
Vinpocetine	0.173	0.042	0.030	0.006*	1.597	0.039	0.028	0.006*
BMSCs	0.007	0.001	0.001	0.004*	1.841	0.072	0.051	0.010*
Vinpocetine+BMSCs	0.054	0.021	0.015	0.005*	2.042	0.040	0.028	0.002*

Furthermore, the expression of the mRNA of PEDF was assessed in the ARPE-19 cells and was found to be increased by exposure to 10 μM Aβ1-42. In contrast to the VEGF-A response, subsequent treatment with vinpocetine, BM-MSCs, and their combination (vinpocetine+BMSCs) led to a further significant increase in PEDF mRNA expression, indicating an anti-angiogenic response (Figure 3B). 

Visual cycle gene expression

**Figure 4:**
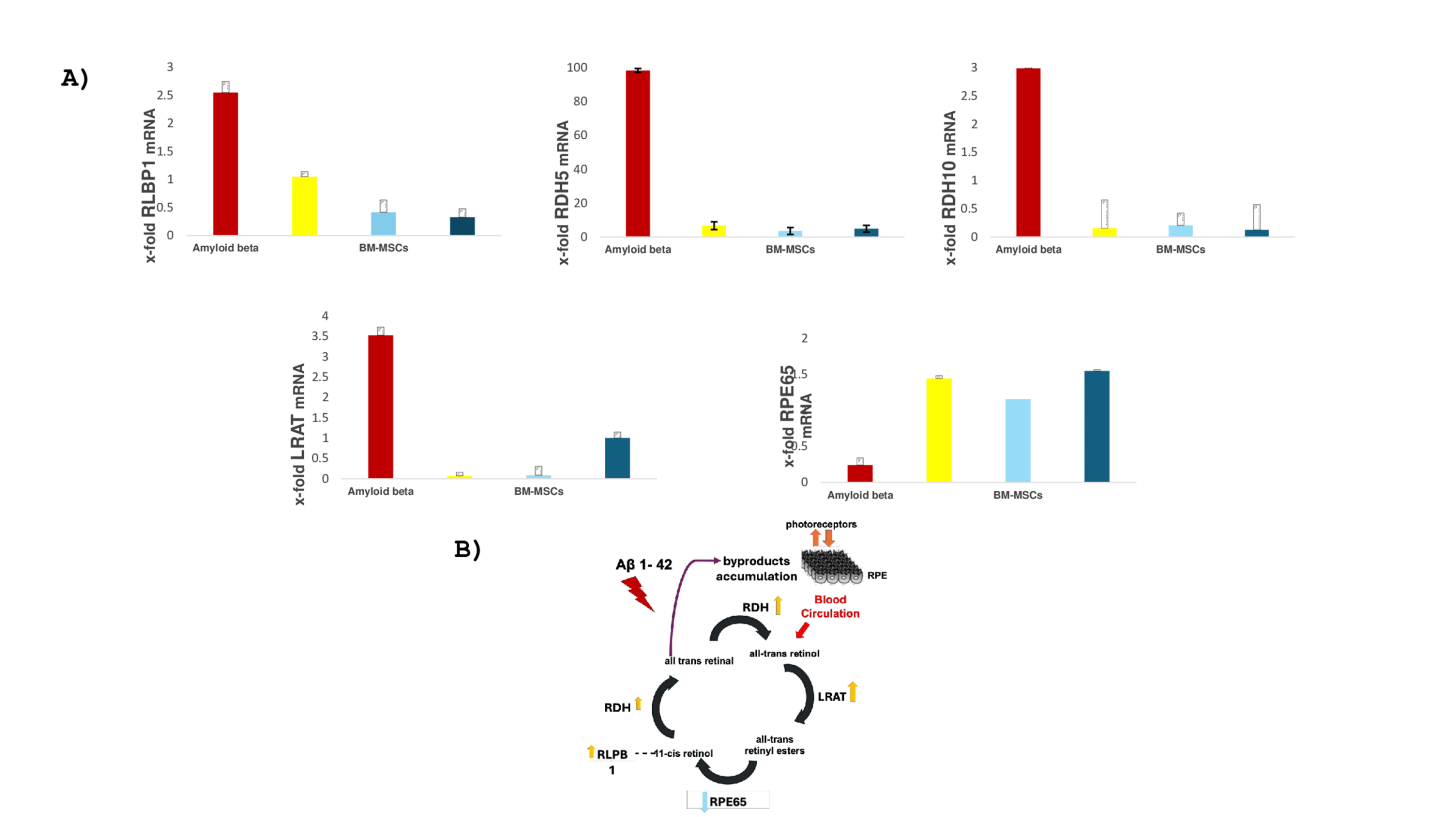
BMSCs and vinpocetine restore visual cycle gene balance in Aβ₁₋₄₂-treated ARPE-19 cells A) Visual cycle gene transcription in ARPE 19 across different groups of intervention. Visual cycle genes transcriptional level was assessed in the ARPE 19 cell line following exposure to Aβ 1-42 and subsequent treatment with BMSCs, vinpocetine, and their combination. Aβ 1-42 upregulated the expression of LRAT, RDH5, RDH10, and RLPB1 and downregulated the expression of RPE-65, an effect that was reversed by all treatment groups. Data are presented as mean ± standard error, with a minimum of three replicates per condition. * indicated statistical significance B) A proposed model illustrating the effect of Aβ 1-42 on visual cycle gene expression, leading to the accumulation of retinoid byproducts that disrupt the normal communication between photoreceptors and RPE. BMSCs - bone marrow stem cells; LRAT - lecithin retinol acyltransferase; RDH5 - retinol dehydrogenase 5; RDH10 - retinol dehydrogenase 10; and RLPB1 - retinaldehyde-binding protein 1; RPE - retinal pigment epithelial

The expression of genes related to the visual cycle was determined by real-time RT-PCR. Lecithin retinol acyltransferase (LRAT), retinol dehydrogenase 5 and 10 (RDH5 and RDH10), and retinaldehyde-binding protein 1 (RLPB1) were significantly upregulated following exposure of ARPE-19 to 10 μM Aβ1-42, while the expression of retinoid isomerase (RPE65) was downregulated. Subsequent exposure of ARPE-19 to vinpocetine, to BMSCs, and to their combination (vinpocetine+BMSCs) significantly reversed the effects of Aβ1-42 (Figure 4). 

Morphological changes of ARPE 19 cells across the intervention groups 

**Figure 5:**
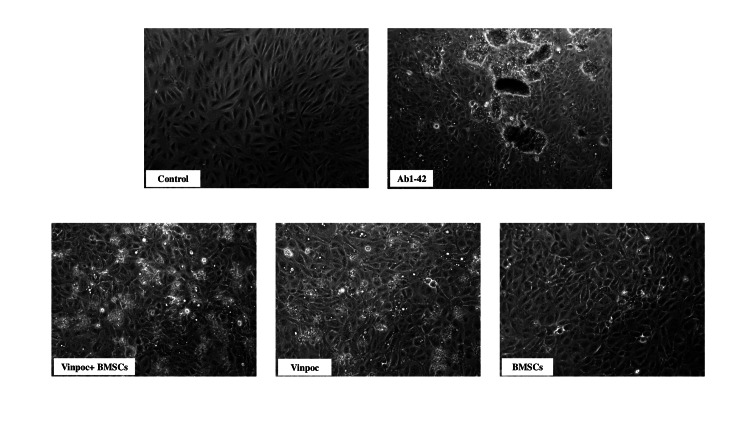
Morphological assessment of ARPE-19 morphology following Aβ 1-42 exposure and treatment Microscope evaluation of the ARPE-19 cell morphology, following Aβ1-42 induced damage and subsequent treatment with vinpocetine, bone marrow stem cells (BMSCs), and their combination. The images were acquired using a 10× objective lens.

Morphological assessment of ARPE-19 cells confirmed the cytotoxic effects of Aβ 1-42, as evidenced by altered cell shape and reduced adherence. Treatment with vinpocetine, BMSCs, and their combination visibly improved cellular morphology, with cell populations exhibiting enhanced structural integrity and strong adherence to the culture surface (Figure 5).

## Discussion

Our study demonstrates that Aβ 1-42 alters the transcriptional level of key visual cycle genes, promoting the accumulation of retinoid byproducts known to impair photoreceptors-RPE interactions [18]. Similar observations have been previously reported, supporting an age-related up-regulation of the main visual cycle genes [19]. Furthermore, our findings corroborate previous findings that Aβ 1-42 induces both cytotoxicity and angiogenesis [6,15]. Notably, our data reveal that treatment with BMSCs, vinpocetine, and their combination restores RPE cell viability, attenuates angiogenic signaling, and reverses the Aβ-induced transcriptional dysregulation of the visual cycle. Collectively, these findings support the potential of combined BMSCs and vinpocetine in addressing the underlying pathology of AMD and propose a novel mechanism through which Αβ 1-42 may contribute to retinal degeneration via dysregulation of the visual cycle.

Specifically, our findings reveal that Aβ 1-42 alters visual cycle gene expression, highlighting a novel therapeutic target in AMD pathogenesis. The accumulation of visual cycle byproducts, particularly A2E, has been shown to increase RPE susceptibility to blue light-induced photodamage, providing a mechanistic link to AMD development [19]. Previous studies indicate that both light and mechanical stress downregulate visual cycle gene expression through retina-derived signaling, suggesting a protective response by the RPE [20]. Supporting previous transcriptomic data from senescence-accelerated rat models, which reported an unexpected upregulation of phototransduction-related genes, our study similarly found upregulation of key visual cycle genes following Aβ 1-42 exposure, with the exception of RPE65, which was downregulated. Thus, it is speculated that endogenous stressors such as Aβ oligomers can alter visual cycle activity in the RPE and influence 11-cis-retinal synthesis, thereby contributing to retinal pathology.

With respect to retinal neovascularization, emerging evidence indicates that drusen components can promote angiogenesis and progression from dry to neovascular AMD. Drusen accumulation, often occurring years before clinical manifestation, disrupts the delicate balance between pro- and anti-angiogenic factors in the outer retina [19]. Among drusen constituents, Aβ 1-42, a highly toxic isomer, has been implicated in both Alzheimer's disease and retinal degeneration [13]. Aβ 1-42 is produced by retinal ganglion cells and RPE, and has been shown to induce inflammation, mitochondrial dysfunction, and angiogenesis in RPE cells [13]. 

Our study indicates that Aβ 1-42 exposure increases VEGF-A expression, while only modestly upregulating PEDF, thereby creating a pro-angiogenic environment. This aligns with previous studies demonstrating Aβ-induced VEGF expression in RPE cells and its dose-dependent effects on angiogenesis [6]. Specifically, researchers demonstrated that the expression of VEGF-A in ARPE-19 cells was enhanced after exposure to Aβ, and higher concentrations of Aβ led to elevated levels of VEGF expression [6]. Previously, Yoshida and colleagues discovered that when RPE cells were exposed to Aβ, the amount of VEGF in their conditioned medium increased in a dose-dependent manner [21].

VEGF-A is the main angiogenenic signal in AMD and promotes the proliferation of RPE cells and the formation of choroidal neovascularization [22], whereas PEDF, a serine protease inhibitor initially recognized in RPE cells, has now been shown to exhibit anti-angiogenic, neuroprotective, and anti-apoptotic effects [6]. The interplay between VEGF and PEDF is crucial in maintaining retinal vascular homeostasis [23]. Reduced PEDF levels have been observed in patients with AMD, particularly in the choroid-RPE interface, facilitating the development of choroidal neovascularization (CNV) [24]. Our data suggest that enhancing PEDF expression, particularly via BMSCs and vinpocetine, could be a promising intervention.

Current anti-VEGF therapies for wet AMD, though effective, require repeated administration and pose risks such as endophthalmitis and systemic complications [25]. Thus, safer and longer-lasting treatments are urgently needed. BMSCs have shown regenerative and anti-angiogenic potential in retinal disease models and early clinical trials [10,26]. Similarly, vinpocetine, exhibiting neuroprotective and anti-inflammatory properties, has been shown to inhibit Aβ-induced NF-κB activation and reduce VEGF expression in ARPE-19 [27]. In a retrospective analysis examining the effect of vinpocetine infusion in 280 patients with ocular disorders, researchers concluded that the best results were achieved in cases of macular degeneration [28], supporting its possible role in the management of AMD.

To our knowledge, our findings provide the first evidence of vinpocetine's anti-angiogenic effects in RPE cells and support the combined use of vinpocetine and BMSCs as a novel approach in addressing pathological pathways related to AMD. Prior research has demonstrated that vinpocetine reduces neointimal hyperplasia and pathological vascular remodeling, in part via inhibiting the production of reactive oxygen species (ROS) and ERK1/2 activation in smooth muscle cells [29]. Regarding Aβ-related pathology, our study found that Aβ 1-42 induces a "surprising" upregulation of visual cycle genes; however, RPE65 expression was downregulated. This selective suppression may represent an intrinsic protective mechanism aimed at reducing stressor-induced retinoid cycling and limiting toxic byproduct accumulation. Interestingly, increasing evidence supports the therapeutic potential of pharmacological strategies that slow the visual cycle to prevent retinoid byproduct buildup, which is a key contributor to the development of dry AMD and geographic atrophy [30]. Visual cycle modulators (VCMs), such as fenretinide, a synthetic retinoid that reduces systemic retinol level, are currently under investigation for this purpose [31]. Furthermore, BMSCs, vinpocetine, and their combination significantly restored the altered transcriptional profile in the RPE. However, this in vitro study is limited by the absence of choroidal endothelial components, which also play a significant role in CNV, and the absence of photoreceptors, which modulate the visual cycle. Hence, the translational applicability of the findings remains to be fully established through in vivo validation. Furthermore, while the concentration of DMSO used as a vehicle is within the range commonly employed in vitro, it may still exert off-target effects that could potentially confound the interpretation of the results. Additionally, the use of three biological replicates per condition, while standard in many studies, is considered the minimal threshold for differential expression analysis and may limit the detection sensitivity, particularly for genes with modest expression changes. Future in vivo studies are warranted to validate these findings and to investigate potential synergistic interactions between BMSCs and vinpocetine within a physiologically relevant microenvironment.

## Conclusions

Conclusively, this study demonstrates that Aβ 1-42 contributes to cytotoxicity, angiogenesis, and dysregulation of visual cycle gene expression in retinal pigment epithelial cells, processes that are closely associated with the pathophysiology of AMD. Treatment with BMSCs, vinpocetine, and their combination effectively mitigated these adverse effects in vitro. These findings suggest a potential therapeutic avenue for AMD, warranting further investigation in preclinical and clinical settings.
